# The Auxin Response Factor *TaARF18-A* Negatively Regulates Salt Tolerance in Common Wheat (*Triticum aestivum* L.)

**DOI:** 10.3390/plants15091375

**Published:** 2026-04-30

**Authors:** Yuzhe Wen, Yiying Li, Shuguang Bao, Gaoyi Cao, Ming Li, Junbin Wang, Bo Ding, Xiaodong Xie, Lina Qiu

**Affiliations:** 1 Tianjin Key Laboratory of Intelligent Breeding of Major Crops, International Joint Center for the Mechanismic Dissection and Genetic Improvement of Crop Stress Tolerance, College of Agriculture & Resources and Environmental Sciences, Tianjin Agricultural University, Tianjin 300392, China; 2 College of Basic Sciences, Tianjin Agricultural University, Tianjin 300392, China

**Keywords:** wheat, auxin response factor (ARF), *TaARF18-A*, salt stress, BSMV-VIGS

## Abstract

Soil salinization is one of the major abiotic stresses that influences agricultural production and the environment. Auxin response factors (ARFs) are key components of the auxin signal transduction pathway, while their role in wheat salt stress responses remains unclear. In this study, we identified *TaARF18* as a negative regulator of salt tolerance in wheat. The coding sequences of *TaARF18-A*, *TaARF18-B*, and *TaARF18-D* were 2106, 2088, and 2088 bp, respectively. TaARF18 is a hydrophilic protein featuring typical Auxin-resp and B3 DNA-binding domains and exhibits relatively high evolutionary conservation among Poaceae species. The expression of *TaARF18* was upregulated under salt stress. TaARF18 predominantly accumulated in the nucleus. Silencing of *TaARF18* via the BSMV-VIGS approach enhanced salt tolerance in wheat seedlings. In addition, haplotype analysis based on resequencing data from 355 wheat accessions identified 25, 31, and 16 haplotypes for *TaARF18-A*, *TaARF18-B*, and *TaARF18-D*, respectively. Fourteen wheat accessions carrying different haplotypes were evaluated under salt stress, and *HapIII* of *TaARF18-A* exhibited the highest level of salt tolerance, which can act as a strong selection locus in global wheat breeding. Our findings provide insight into the function of ARFs in salt stress responses and offer a potential target for CRISPR/Cas-mediated salt-tolerant wheat breeding programs.

## 1. Introduction

Common wheat (*Triticum aestivum* L.) is one of the most important staple crops, providing nearly 18% of the calories and 19% of the protein for human consumption [1]. With the growing global population, the demand for wheat will increase substantially [2]. Abiotic stresses such as salinity pose a severe threat to wheat production and quality [3]. Approximately 1 billion hectares of land worldwide are currently affected by salinization, representing about 33% of irrigated lands. Moreover, due to population growth, climate change and unreasonable irrigation methods, soil salinization is being severely exacerbated [4,5]. Consequently, mining salt-tolerant genes and employing them to develop salt-tolerant wheat is a practical and economic way to utilize salt-affected land.

Auxin is one of the earliest discovered and most important plant hormones. It regulates a wide range of physiological processes, including phototropism, geotropism, vascular tissue formation, root growth, and flower and fruit development [6,7,8]. Auxin response factors (ARFs), a critical class of transcription factors that serve as the core components of the auxin signal transduction pathway, are involved in plant responses to abiotic stresses [9]. Accumulating evidence indicates that ARFs participate in abiotic stress responses in multiple plant species. In rice, *OsARF12* acts as a transcriptional activator that regulates the expression of *OsSOS1* and *OsHKT1;5*, thereby enhancing salt tolerance; additionally, the *OsIAA8*-*OsARF12* module modulates drought stress responses [10,11], while *OsARF18* increases rice sensitivity to salt [12]. In maize, *ZmARF1* contributes to salt stress tolerance in transgenic *Arabidopsis* [13]. In tomato, *SlARF2* and *SlARF4* negatively regulate salt tolerance [14,15]. Overexpression of sweet potato *ARF5* significantly enhances salt and drought tolerance in transgenic *Arabidopsis* [16]. In wheat, *TaARF15-A.1* participates in root and leaf development, *TaARF15-A1* negatively regulates senescence, and *TaARF8*, *TaARF9* and *TaARF21* respond to cold stress [17,18]. However, the roles of ARFs in wheat salt stress responses remain poorly understood.

In rice, *OsARF18* represses the expression of the gene encoding asparagine synthetase 1 (OsAS1), loss of *OsARF18* function leads to increased *OsAS1* expression, which enhances nitrogen (N) utilization efficiency by promoting asparagine production and preventing excessive accumulation of ammonium (NH_4_^+^) in shoots, ultimately reducing yield loss in saline soils and improving grain yield even under normal conditions [12]. To investigate the role of auxin response factors (ARFs) in wheat salt tolerance, we cloned *TaARF18* and employed barley stripe mosaic virus–virus-induced gene silencing (BSMV-VIGS) to validate its function under salt stress, revealing that it enhances wheat salt tolerance. Furthermore, haplotype analysis combined with salt tolerance assessment identified *HapIII* of *TaARF18-A* as the optimal haplotype, highlighting its value as a genetic resource for global wheat breeding. These findings deepen our understanding of ARF functions and provide a promising target for developing salt-tolerant wheat varieties.

## 2. Results

### 2.1. Cloning and Characterization of the Wheat ARF Gene TaARF18

**Figure 1:**
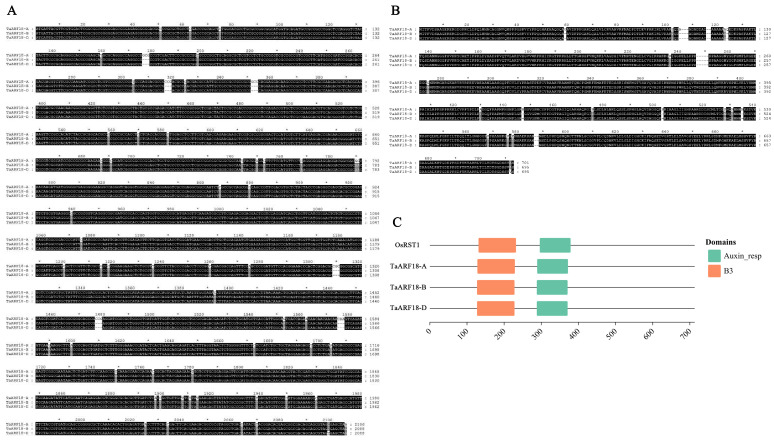
Cloning and sequence characterization analysis of *TaARF18* in wheat. (**A**)Nucleotide sequence alignment of *TaARF18-A*, *TaARF18*-B, and *TaARF18-D*. Identical nucleotides are indicated by a black background, partially identical sites by a gray background, and different nucleotides by a white background. (**B**) Amino acid alignment of TaARF18. Identical amino acid residues are indicated by a black background, similar residues by a gray background, and different residues by a white background. (**C**) Conserved domain analysis of TaARF18 and OsARF18 (OsRST1) proteins. *: Asterisks indicate position markers in the sequence alignment.

To identify the *TaARF18* homoeologous genes in wheat and characterize their sequence features and evolutionary relationships, rice *ARF18* sequences were used as queries to search the wheat genome database (IWGSC RefSeq v1.0) in EnsemblPlants (http://plants.ensembl.org/index.html, accessed on 1 June 2025). Three homoeologs of wheat *TaARF18* located on chromosomes 7A, 7B, and 7D were identified and designated as *TaARF18-A*, *TaARF18-B*, and *TaARF18-D*, respectively, based on their chromosomal positions (Table S3). The sequence length of *TaARF18-A* was 2106 bp, whereas those of *TaARF18-B* and *TaARF18-D* were both 2088 bp. The coding sequences (CDSs) of the three *TaARF18* homoeologs were highly conserved; sequence alignment revealed that *TaARF18-A* shared 98.5% sequence similarity with both *TaARF18-B* and *TaARF18-D*, whereas *TaARF18-B* and *TaARF18-D* exhibited 99.9% similarity and differed by only one nucleotide, indicating a higher sequence consistency between B and D subgenome homoeologs (Figure 1A). *TaARF18-A*, *TaARF18-B*, and *TaARF18-D* encode 701, 695, and 695 amino acid residues, respectively. Alignment of deduced amino acid sequences showed that the three TaARF18 homoeologs shared 97.44% sequence similarity (Figure 1B). Conserved domain analysis indicated that ARF18/ARF18-like proteins from rice and TaARF18 proteins from wheat both contain the characteristic Auxin-resp domain and the B3 DNA-binding domain (Figure 1C). The positions and lengths of these two domains were highly consistent between rice and wheat, indicating a high degree of evolutionary conservation of this gene across species.

To characterize the TaARF18 proteins, we used the ExPASy server to predict their physicochemical properties. The molecular weight and theoretical isoelectric point (pI) of TaARF18-A were 75.79 kDa and 8.15, respectively, with alanine (Ala), arginine (Arg), and leucine (Leu) accounting for 10.6%, 6.6%, and 7.7% of the amino acid composition, respectively. TaARF18-B and TaARF18-D exhibited similar molecular weights (75.21 kDa and 75.24 kDa, respectively) and identical theoretical pI values (7.90), with comparable amino acid compositions—Ala comprising approximately 10% and Arg and Leu present at similar proportions. Among TaARF18-A, TaARF18-B, and TaARF18-D, the numbers of negatively charged residues, aspartate (Asp) and glutamate (Glu) were 71, 70, and 70, respectively, while the numbers of positively charged residues, Arg and lysine (Lys) were 74, 72, and 72, respectively, indicating similar net charge properties across the three homoeologs. Hydropathy analysis of the proteins encoded by the three *TaARF18* homoeologs revealed highly similar hydropathy profiles, and the grand averages of hydropathicity (GRAVY) values were all negative, suggesting that these proteins are hydrophilic (Figure S1A–C). Additionally, TMHMM 2.0 analysis was performed to assess the presence of transmembrane domains, which revealed no transmembrane helices in any of the three proteins (Figure S1D–F).

### 2.2. Secondary and Tertiary Structures of TaARF18 Protein

**Figure 2:**
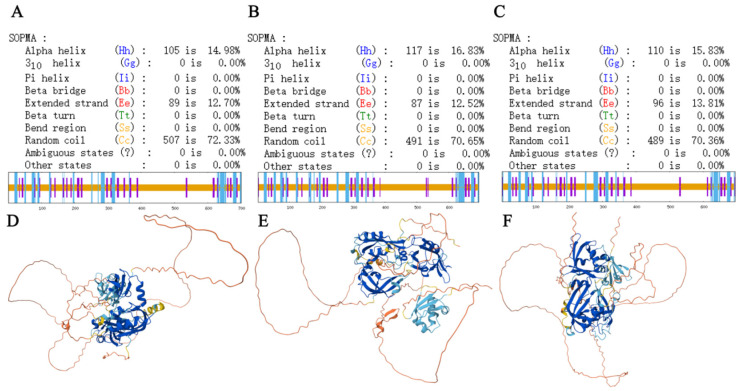
Secondary and tertiary structures of TaARF18 proteins. (**A**–**C**) Secondary structures of TaARF18-A (**A**), TaARF18-B (**B**), and TaARF18-D (**C**). (**D**–**F**) Predicted three-dimensional structures of TaARF18-A (**D**), TaARF18-B (**E**), and TaARF18-D (**F**). Colors indicate the pLDDT confidence score predicted by AlphaFold3, with dark blue representing very high confidence, light blue representing confident regions, yellow representing low confidence, and orange representing very low confidence or potentially disordered regions.

To further investigate the structural features of TaARF18, SOPMA and AlphaFold were employed to predict its secondary and tertiary structures. Secondary structure predictions indicated that TaARF18-A, TaARF18-B, and TaARF18-D were predominantly composed of random coils (72.33%, 70.65%, and 70.36%, respectively), followed by α-helices (14.98%, 16.83%, and 15.83%, respectively) and extended strands (12.70%, 12.52%, and 13.81%, respectively); no β-turns were detected in any homoeolog (Figure 2A–C). Subsequently, tertiary structure modeling revealed that all three TaARF18 proteins adopt a relatively loose overall conformation, featuring multiple helical regions and pronounced chain-like structural elements (Figure 2D–F).

### 2.3. Analysis of the cis-Acting Element in the Promoters of TaARF18 Homoeologs

To investigate the potential transcriptional regulation of *TaARF18* in response to abiotic stress and environmental stimuli, cis-acting element analysis of the promoter regions of the *TaARF18* homoeologs was performed using the web-based PlantCARE tool. Five major categories of cis-acting regulatory elements were identified in the promoter regions of all three *TaARF18* homoeologs: core promoter elements, light-responsive elements, hormone-responsive elements, stress-related elements, and development-associated elements. Notably, several cis-elements associated with abiotic stress and environmental responses were detected, including motifs associated with low-temperature responsiveness, drought inducibility, defense and stress responsiveness, anaerobic induction, and light regulation (Figure S2). For example, MYB-binding sites implicated in drought inducibility were present, while light-responsive elements and MYBHv1-binding sites suggested potential regulation of *TaARF18* under changing light conditions.

### 2.4. Phylogenetic Analysis of TaARF18

**Figure 3:**
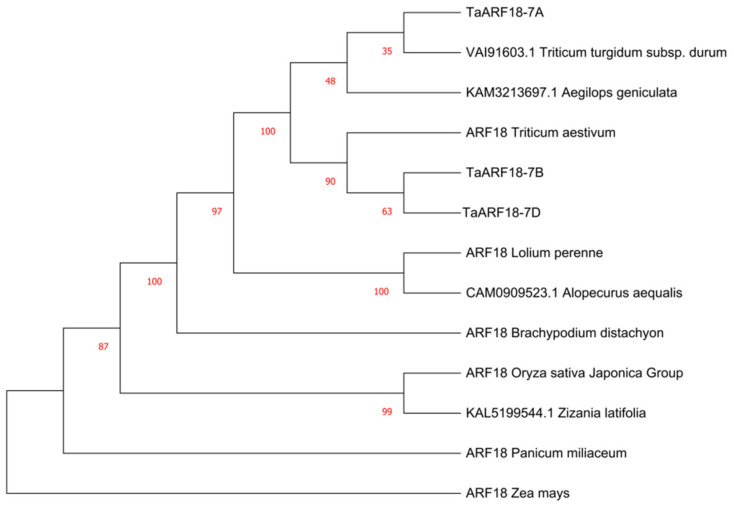
Phylogenetic analysis of TaARF18.

To analyze the phylogenetic relationship of TaARF18 among different species, a neighbor-joining (NJ) tree was constructed using ARF protein sequences from *Triticum turgidum*, *Aegilops*, *Triticum aestivum*, *Lolium perenne*, *Alopecurus aequalis*, *Brachypodium distachyon*, *Oryza sativa*, *Zizania latifolia*, *Panicum miliaceum*, and *Zea mays*. The results indicated that the wheat TaARF18 and ARF18 (or ARF18-like) proteins from eight gramineous species were divided into two major clades (Figure 3). TaARF18-A clustered with common wheat (Chinese Spring, *Triticum aestivum*), whereas TaARF18-B and TaARF18-D formed a separate clade and grouped with *Aegilops tauschii*. Moreover, the three wheat TaARF18 proteins clustered within a larger group together with that from barley (*Hordeum vulgare*), *Brachypodium distachyon*, and rice (*Oryza sativa*), suggesting that ARF18 exhibits relatively conserved evolutionary relationships among Poaceae species.

### 2.5. TaARF18 Responses to Salt Stress

**Figure 4:**
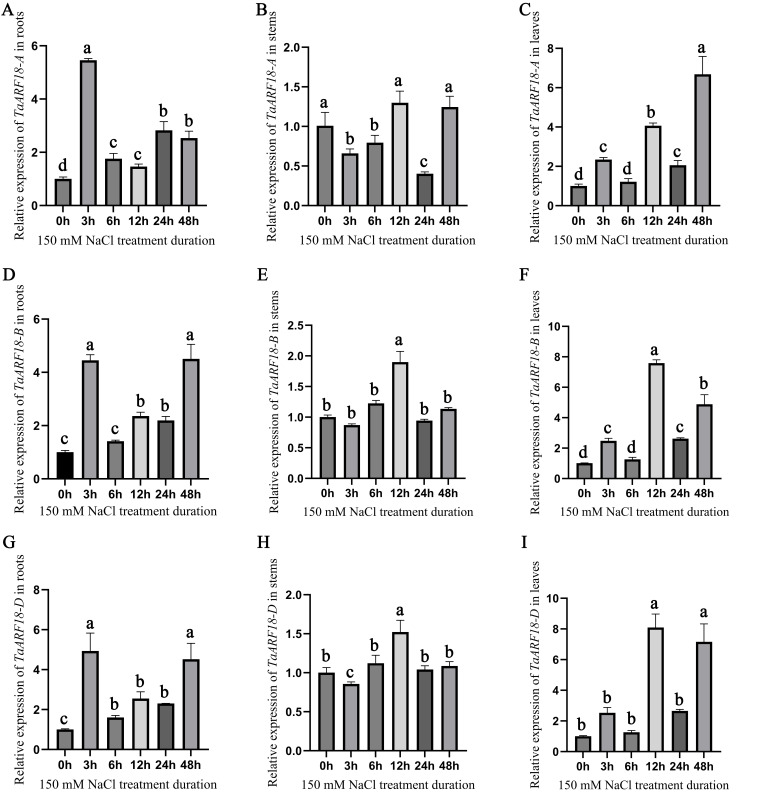
Expression patterns of *TaARF18* in response to salt stress in wheat. (**A**–**I**) Relative transcript levels of *TaARF18-A*, *TaARF18-B*, and *TaARF18-D* in roots, stems, and leaves at the indicated time points after 150 mM NaCl treatment. Different lowercase letters indicate significant differences among time points (one-way ANOVA, *p* < 0.05).

To investigate the effects of salt stress on *TaARF18* expression, the spatial and temporal expression patterns of its homoeologs under normal and salt stress conditions were analyzed by quantitative reverse transcription PCR (qRT-PCR). The results indicated that, overall, the transcript abundance of *TaARF18* increased following salt stress, with expression patterns varying among seedling roots, shoots and leaves. Responses were stronger in roots and leaves than in stems. In roots, *TaARF18-A* peaked rapidly at 3 h (approximately 5–6-fold) and was re-induced at 24–48 h, whereas *TaARF18-B* and *TaARF18-D* exhibited similar early (3 h) and late (48 h) upregulation (approximately 4–5-fold). Stem responses were relatively modest, *TaARF18-A* remained largely unchanged, while *TaARF18-B/D* reached a moderate maximum at 12 h (approximately 1.5–2-fold). In leaves, all three homoeologs were strongly induced, with *TaARF18-A* peaking at 48 h (approximately 6–7-fold) and *TaARF18-B/D* showing their highest induction at 12 h (approximately 7–8-fold) and remaining elevated at 48 h (Figure 4). These results suggest that *TaARF18* is involved in tissue-specific salt stress responses, particularly rapid transcriptional activation in roots and strong, sustained induction in leaves.

### 2.6. Subcellular Localization of TaARF18

**Figure 5:**
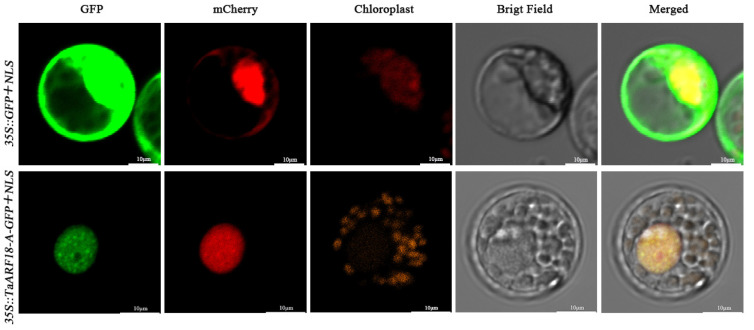
Subcellular localization of TaARF18-A in wheat protoplasts. *35S::GFP*+*NLS* (control) and *35S::TaARF18-A-GFP*+*NLS* recombinants were transiently expressed in wheat protoplasts. Green indicates GFP signals, and red indicates RFP signals. Results were observed after transformation for 14 h with confocal microscopy. Scale bars = 10 μm.

To determine the subcellular localization of TaARF18-A, the *TaARF18-A-GFP* fusion construct was transiently expressed in wheat protoplasts, with the empty GFP vector and a nuclear localization signal (NLS) marker serving as controls. The GFP control signal was detected in both the nucleus and cytoplasm, whereas TaARF18-A-GFP predominantly accumulated in the nucleus and exhibited strong overlap with the NLS marker (Figure 5). These results indicate that TaARF18-A is a nucleus-localized protein.

### 2.7. Silencing of TaARF18 Increases the Salt Tolerance of Wheat Seedlings

**Figure 6:**
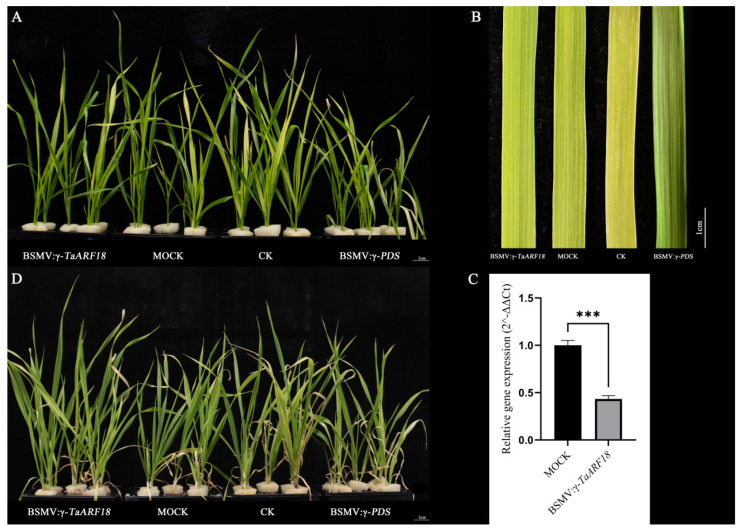
BSMV-VIGS-mediated silencing of *TaARF18* and phenotypes of wheat seedlings under salt stress. (**A**) Phenotypes of BSMV-infected plants before salt treatment. (**B**) Phenotypes of wheat leaves after BSMV inoculation. (**C**) The transcript levels of *TaARF18* in *BSMV:γ-TaARF18* plants and MOCK plants. Values are means ± SDs (n = 3). Significance was determined by Student’s *t*-test (*** *p* < 0.001). (**D**) Phenotypes of successfully BSMV-infected and non-inoculated plants after 12 days of salt treatment. MOCK, *BSMV:γ-NLR1*; CK, non-inoculated plants; *BSMV:γ-PDS*, positive control for viral infection. Scale bars indicate 2 cm (**A**,**D**) and 1 cm (**B**).

To investigate the role of *TaARF18* in salt stress response in wheat seedlings, the barley stripe mosaic virus–virus-induced gene silencing (BSMV-VIGS) system was employed to silence *TaARF18* and validate its function. Wheat seedlings with uniform growth were selected and inoculated with BSMV. Silencing of *TaARF18* had no adverse effect on seedling growth (Figure 6A), and white streaks were observed in leaves infected with *BSMV-γ-PDS*, indicating that the VIGS system functioned effectively (Figure 6B). Subsequent qRT-PCR analysis revealed that *TaARF18* transcript levels were significantly reduced in silenced plants, confirming successful gene silencing (Figure 6C). Following treatment with 150 mM NaCl, *TaARF18*-silenced plants exhibited less leaf curling and chlorosis than control plants after 12 days of salt stress (Figure 6D).

**Figure 7:**
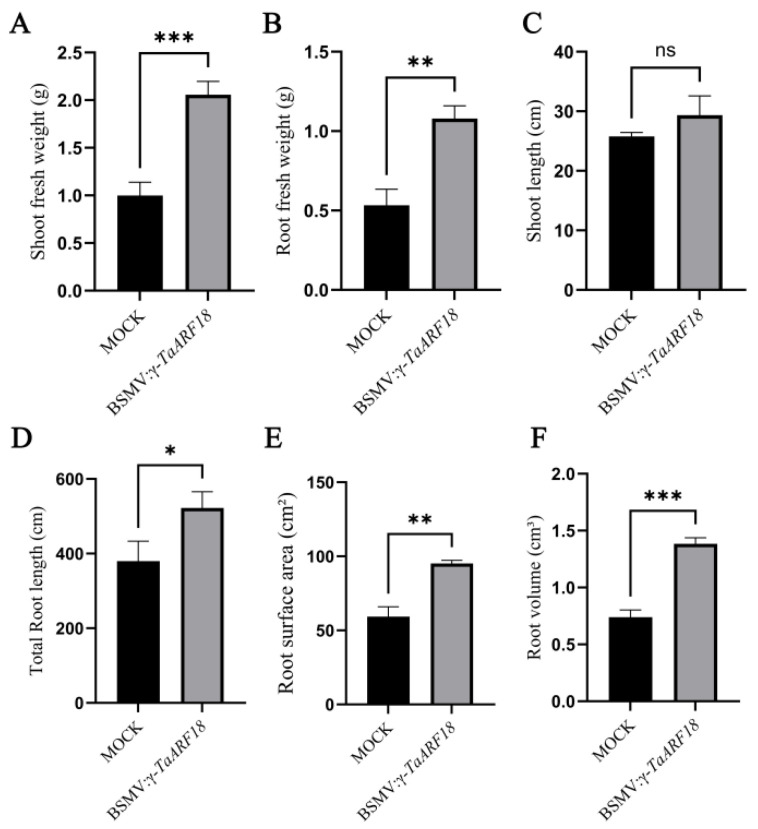
Morphological parameters of *TaARF18*-silenced and mock seedlings under salt stress. (**A**) Shoot fresh weight. (**B**) Root fresh weight. (**C**) Shoot length. (**D**) Total root length. (**E**) Root surface area. (**F**) Root volume. Data are presented as mean ± SD (n = 3). Statistical significance was determined using Student’s *t*-test; ns, not significant; * *p* < 0.05, ** *p* < 0.01, *** *p* < 0.001.

To further characterize the phenotypic differences between *TaARF18*-silenced and control plants, several morphological parameters were measured. The results indicated that shoot fresh weight, root fresh weight, total root length, root surface area, and root volume were significantly increased in *TaARF18*-silenced plants, whereas shoot length showed no significant difference compared with control plants (Figure 7A–F). Root scanning images further revealed that *TaARF18*-silenced plants developed a more robust root system with an increased number of lateral roots relative to mock-inoculated plants (Figure S3).

**Figure 8:**
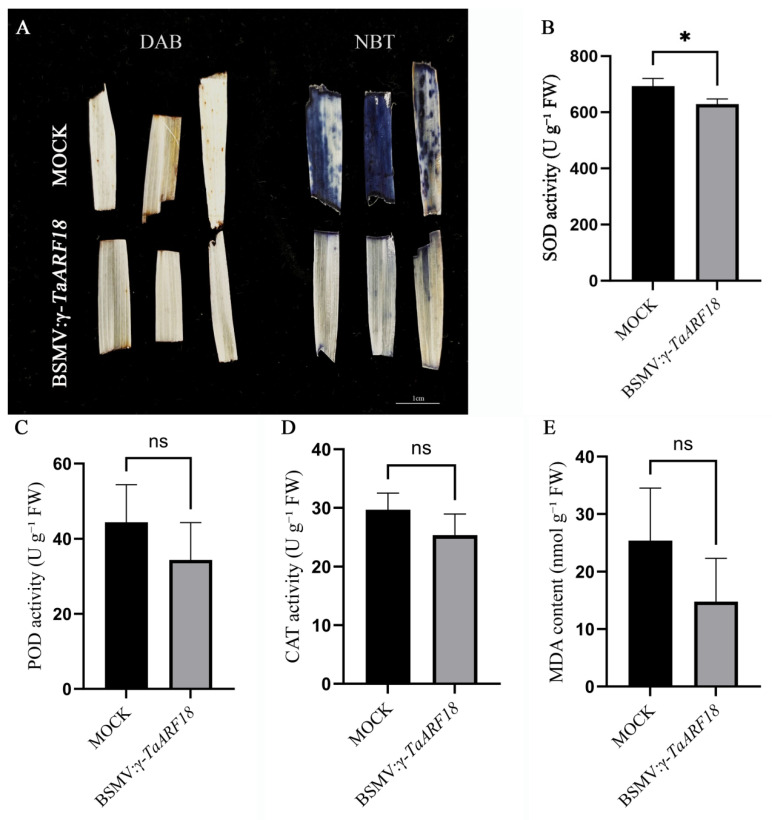
The effect of silencing *TaARF18* on the antioxidant accumulation in the leaves of wheat seedlings under salt stress. (**A**) Histochemical staining of leaves with DAB and NBT to visualize the accumulation of H_2_O_2_ and O^2−^ in the leaves of wheat seedlings, respectively. (**B**–**D**) The activities of SOD, POD, and CAT in wheat seedling leaves, respectively. (**E**) MDA content in wheat seedling leaves. Data are presented as mean ± SD (n = 3). Statistical significance was determined using Student’s *t*-test; ns, not significant; * *p* < 0.05. Scale bar = 1 cm.

To assess the effect of *TaARF18* on antioxidant capacity in wheat leaves under salt stress, 3,3-diaminobenzidine (DAB) and nitro blue tetrazolium (NBT) staining methods were used to measure the content of H_2_O_2_ and O^2−^ in leaves, respectively. The results showed H_2_O_2_ and O^2−^ were lower in *TaARF18*-silenced plants compared with mock plants (Figure 8A). Consistently, SOD activity was significantly lower in *TaARF18*-silenced plants than mock plants, whereas POD and CAT activities and MDA content showed an overall decreasing trend (Figure 8B–E). Collectively, the results demonstrated that silencing of *TaARF18* could enhance growth maintenance and mitigate oxidative stress accumulation under salt stress conditions.

### 2.8. Haplotypes of TaARF18 and Salt Tolerance Evaluation of Haplotyped Materials

In order to analyze the population-scale variations in the *TaARF18* (*A/B/D*) coding sequence (CDS) regions, we retrieved whole-genome resequencing data for 355 common wheat accessions from the Wheat Omics database and used TBtools to conduct haplotype analysis of the three *TaARF18* homoeologs. We identified 25, 31, and 16 haplotypes for *TaARF18-A*, *TaARF18-B*, and *TaARF18-D*, respectively (Figure S4, Table S4).

**Figure 9:**
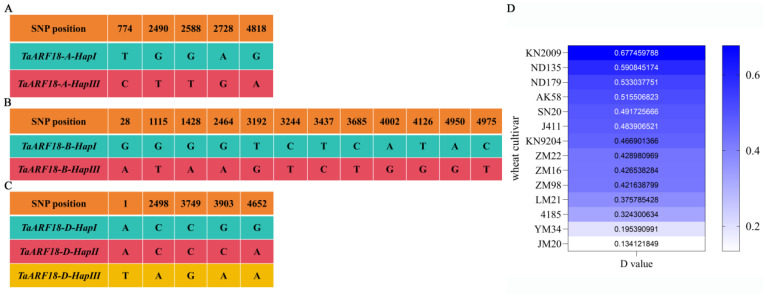
Haplotype variation and salt tolerance evaluation of 14 wheat cultivars. (**A**–**C**) Haplotypes of *TaARF18-A* (**A**), *TaARF18-B* (**B**), and *TaARF18-D* (**C**) based on their coding regions in 14 wheat accessions. (**D**) Heatmap of the integrated salt tolerance index (D value) for the 14 cultivars.

**Figure 10:**
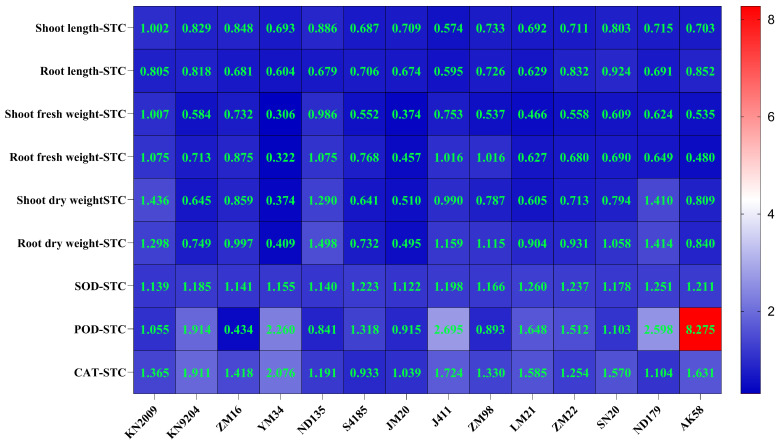
Heatmap of salt tolerance coefficients (STCs) for growth and physiological traits in 14 wheat cultivars.

To evaluate the association between haplotypes and salt tolerance, 14 wheat cultivars with different haplotypes were treated with 150 mM NaCl at the two-leaf stage (Figure 9A–C, Table S1). Growth indicators (e.g., shoot length, root length, shoot fresh weight, root fresh weight, shoot dry weight, and root dry weight) and the activities of SOD, POD, and CAT were measured after 15 days, and the salt tolerance coefficient (STC) was then calculated for each trait (Figure 10). Subsequently, a comprehensive D value was derived using membership function normalization and weight assignment (Tables S5 and S6). Among the 14 cultivars tested, the third haplotype of *TaARF18-A* exhibited the highest D value, indicating the strongest salt tolerance (Figure 9D).

## 3. Discussion

Salinity is one of the most severe environmental hazards for wheat growth and grain yield worldwide [19]. Identifying salt-tolerant genes/loci and leveraging them to develop salt-tolerant crop varieties represents the most economical and effective strategy for utilizing salinized land. In this study, the fresh weight of shoots and roots, total root length, root surface area, and root volume of barley stripe mosaic virus (BSMV)-induced silencing lines were significantly higher than those of mock-inoculated lines 12 days after treatment with 150 mM NaCl (Figure 7), indicating that *TaARF18* negatively regulates salt tolerance in wheat. In recent years, CRISPR/Cas genome editing systems have rapidly emerged as a powerful tool for gene function validation, gene therapy, and plant breeding [20]. Furthermore, an increasing number of gene editing platforms with independent intellectual property rights have been developed [21,22,23,24,25], and genes that can enhance the efficiency of genetic transformation have also been identified [26,27]. The use of immature haploid embryos for genetic transformation has enabled the rapid genetic stabilization of transgenic and genome-edited wheat [28]. Additionally, genome editing for biosafety purposes has received regulatory approval in China. Therefore, *TaARF18* may serve as a potential target for improving the salt tolerance of wheat and could be utilized in gene editing breeding.

It has been reported that salt stress could trigger excessive accumulation of reactive oxygen species (ROS), such as hydroxyl radicals, H_2_O_2_, and superoxide anions, which increase MDA content in plants [29,30]. Enzymatic systems, such as POD, SOD and CAT, play crucial roles in scavenging ROS, and an increase in their activity can reduce ROS accumulation and prevent oxidative damage [31,32,33]. In this study, we found that the levels of H_2_O_2_ and superoxide anion (O_2_^−^) in mock plants were higher than those in *TaARF18*-silenced lines (Figure 8A). Correspondingly, under salt stress, no significant differences were observed between mock and *TaARF18*-silenced lines in POD and CAT activities and MDA content, with the exception of SOD activity (Figure 8B–E). This may be attributed to prolonged exposure to salt stress (12 days). In contrast, after 12 days of salt stress, *TaRS15-3B* overexpression lines exhibited a highly significant increase in POD activity and MDA content compared to a wild type [34]. Taken together, these results imply that *TaARF18* may play a role similar to that of *OsARF18* in rice, which negatively regulates rice salt tolerance by repressing the expression of *OsAS1*, a gene that encodes asparagine synthetase 1. Loss of *OsARF18* function leads to increased *OsAS1* expression, enhancing nitrogen use efficiency by promoting asparagine synthesis and preventing excess ammonium accumulation [12]. So, further experiments need to be carried out to verify the hypothesis.

Auxin response factors (ARFs) encode transcription factors that regulate the expression of genes in response to auxin [35]. A growing body of research suggests that ARFs participate in plant stress resistance. For instance, *GmARF16* negatively regulates soybean salt tolerance [36]. Lu et al. found that *AtARF7* and *AtARF9* are essential for salt-induced *SOS1* expression and salt tolerance in *Arabidopsis* [37]. *OsARF12* increases salt tolerance in rice by integrating auxin signaling with ROS scavenging, ionic homeostasis and photosynthetic networks [10]. *IbARF5* is involved in carotenoid biosynthesis and salt and drought tolerance in transgenic *Arabidopsis* [16]. In wheat, Qiao et al. clarified the characterization and expression patterns of ARFs in wheat [17], and Li et al. discovered that *TaARF15* delays wheat senescence [18]. However, the role of ARF in stress response remains elusive. Here, we demonstrated that *TaARF18* negatively regulates wheat salt tolerance; however, to identify the functions of additional ARFs in response to stress, further research is necessary.

Common wheat (*Triticum aestivum* L.) serves as a primary calorie source for billions of people worldwide [38]. It possesses a large, complex, repetitive and allohexaploid genome consisting of A, B, and D subgenomes (2n = 6x = 42, AABBDD). Moreover, it is recalcitrant to transformation, which causes it to lag behind other cereals such as rice and maize [39]. In recent years, significant advances in DNA sequencing technology, data analysis algorithms, and pipelines for assembling genomes have greatly facilitated the release of the genomes of wheat and its wild ancestral species [40]. While the genetic transformation of wheat is relatively difficult, less effective and costly [41], these restrictions facilitated the application of virus-induced gene silencing (VIGS), an effective tool for the rapid generation of gene knockdown phenotypes in plants. It has played a crucial role in validating the functions of genes in wheat, including those responding to abiotic (e.g., salt and drought) and biotic stresses (e.g., powdery mildew, leaf rust, and stripe rust resistance gene), as well as genes that regulate quality traits (e.g., starch synthesis) and seed size [42,43,44,45,46,47,48]. In this study, the VIGS method was employed to verify the role of *TaARF18*, and we identified it as a negative regulator of salt tolerance in common wheat, which provides insights into the functions of ARFs.

## 4. Materials and Methods

### 4.1. Plant Materials and Growth Conditions

The common wheat cultivar Fielder was used as the plant material to clone *TaARF18* and explore the expression pattern of *TaARF18* before (0 h) and at different time points (3, 6, 12, 24, and 48 h) after 150 mM NaCl treatment. Fourteen wheat varieties with different haplotypes were employed to assess salt tolerance (Table S1). Seeds were germinated in germination incubators for 3 days and then transferred to hydroponic containers containing distilled water for further growth. All plants were grown in a growth chamber under a photoperiod of 16 h light/8 h dark at a temperature of 25 °C and a light intensity of 330 µmol m^−2^ s^−1^.

### 4.2. Total RNA Extraction and Reverse Transcription

Total RNA was extracted using a FastPure Universal Plant Total RNA Isolation Kit (Vazyme Biotech, Nanjing, China), and 1 µg of RNA was reverse transcribed to synthesize cDNA using a PrimeScript RT Perfect Real Time Kit (Takara, Dalian, China).

### 4.3. Gene Cloning, Sequencing, and Phylogenetic Analysis of TaARF18

Specific primer pairs were designed to obtain the complete coding sequence (CDS) of *TaARF18-A*, *TaARF18-B*, and *TaARF18-D* (Table S2). The polymerase chain reaction (PCR) was performed using PrimeSTAR Max DNA Polymerase Ver.2 (Takara, Dalian, China). The PCR system contained 25 µL master mix, 2 µL forward and reverse primers each (0.2 µM), 2 µL cDNA (200 ng/µL), and 19 µL nuclease-free water. PCR cycling was as follows: 35 cycles of 98 °C denaturation for 10 s, 56 °C primer annealing for 15 s, and 68 °C extension for 30 s. The PCR products were verified by agarose gel electrophoresis, the target bands were excised and purified by a TaKaRa MiniBEST Agarose Gel DNA Extraction Kit, Ver.4.0 (Takara, Dalian, China), and the purified amplicons were sequenced by Sangon Biotech (Shanghai, China).

The molecular weight, theoretical isoelectric point, and hydrophilicity of TaARF18 were predicted using the ProtParam tool on the ExPASy platform (https://web.expasy.org/protparam/, accessed on 17 October 2025). The transmembrane domains of TaARF18 were predicted using the TMHMM 2.0 online server (https://services.healthtech.dtu.dk/services/TMHMM-2.0/, accessed on 29 October 2025). The SOPMA online analysis website (https://npsa-prabi.ibcp.fr/cgi-bin/npsa_automat.pl?page=npsa_sopma.html, accessed on 22 October 2025) and AlphaFold (https://alphafoldserver.com/, accessed on 24 October 2025) were used to predict the secondary and tertiary structures of TaARF18 protein sequences, respectively. Cis-acting elements analysis of the *TaARF18* promoter regions was performed using the PlantCARE database (http://www.plant-care.com/, accessed on 4 October 2025). The conserved domains of TaARF18 proteins were predicted in the NCBI Conserved Domain Database (CDD) Batch CD-Search tool (https://www.ncbi.nlm.nih.gov/, accessed on 25 October 2025).

The amino acid sequence of TaARF18 was subjected to BLAST analysis in the protein database of NCBI (https://www.ncbi.nlm.nih.gov/, accessed on 18 October 2025), and 10 protein sequences from rice, maize and wheat with higher similarity were downloaded. The protein sequences were aligned using MEGA X [49]. The neighbor-joining (NJ) method was employed to construct the phylogenetic tree, with 1000 bootstrap replicates to assess node reliability.

### 4.4. Quantitative Reverse Transcription PCR (qRT-PCR) Analysis

Quantitative reverse transcription PCR (qRT-PCR) analysis was conducted using SuperStar Universal SYBR Master Mix (Kangwei Century, Beijing, China) in a volume of 20 µL in the QuantStudio 3 Real-Time PCR System (Thermo Fisher Scientific, Waltham, MA, USA). The reactive system contained 10 µL master mix, 1 µL forward and reverse primers each (0.2 µM), 2 µL cDNA (200 ng/µL), and 6 µL nuclease-free water. The reaction parameters were 95 °C for 5 min, 40 cycles of 95 °C for 10 s, 60 °C for 30 s, and 72 °C for 30 s. All of the specific primers are listed in Table S2. Each sample was quantified at least in triplicate and normalized using the *TaActin* gene as the internal reference; the 2^−∆∆Ct^ method was used to calculate the gene transcriptional abundance [50]. The 0 h sample was set to 1.0. Data are presented as mean ± SD (n = 3).

### 4.5. Subcellular Localization of TaARF18

The full-length CDS of *TaARF18-A* with the stop codon removed was fused into a pBI221-EGFP vector with a ClonExpress Ultra One Step Cloning Kit V3 (Vazyme Biotech, Nanjing, China), designated as *35S::TaARF18-A-GFP*. The GFP and *35S::TaARF18-A-GFP* plasmids were separately co-transformed with TF3-mCherry into wheat protoplasts mediated by PEG4000, as described before [51], where TF3-mCherry is a nuclear localization marker [52]. The transformed protoplasts were incubated at 22 °C for 18 h in darkness, after which GFP signals were observed with 488 nm and 543 nm illumination by confocal laser scanning microscopy (LSM700, CarlZeiss, Oberkochen, Germany).

### 4.6. BSMV-VIGS Assay

We used the online tool SGN VIGS to predict the optimal silencing sequence of *TaARF18* (https://vigs.solgenomics.net/, accessed on 3 October 2025). The predicted sequence was amplified and inserted into an *NheI*-digested BSMV-γ to construct the recombinant vector. The BSMV-γ vector carrying the phytoene desaturase (PDS) gene segment (*BSMV-γ-PDS*) was used to evaluate the efficiency of VIGS. *BSMV-γ-NLR1*, a gene that is not present in common wheat, was utilized as a negative control [53]. *BSMV-α*, *BSMV-γ-TaARF18*, *BSMV-γ-PDS*, and *BSMV-γ-NLR1* were linearized with *MluI*; *BSMV-β* was linearized with *SpeI*. Subsequently, these linearized vectors were transcribed in vitro by using T7 Pro Polymerase (SYNTHGENE, Wuxi, China), and 5′-capped BSMV RNA molecules were produced with Cap Analogs (SYNTHGENE, Wuxi, China). The second fully expanded seedling leaves were infected with equal *BSMV-α, BSMV-β* and *BSMV-γ* recombinant vectors, as previously described [54]. The silencing of PDS in wheat leaves was visually confirmed by the appearance of white streaks, indicating that the VIGS system was effective, and we used qRT-PCR to detect the expression levels of *TaARF18* in the leaves. Wheat seedlings used for BSMV inoculation were grown in half-strength Hoagland’s solution throughout; after confirming gene silencing, the medium was replaced with half-strength Hoagland’s supplemented with 150 mM NaCl, and seedlings were maintained for 12 days for phenotypic observation.

### 4.7. Determination of Root Morphological Indicators

The root systems of wheat seedlings were scanned and analyzed by using WinRHIZO (Shandong Huoerd Electronic Technology Co., Weihai, China). Root images were obtained after scanning, and total root length, root surface area, and root volume were measured.

### 4.8. Histochemical Staining

Hydrogen peroxide (H_2_O_2_) and superoxide anions (O^2−^) were detected using 3,3-diamino-benzidine staining (DAB) and nitroblue tetrazolium (NBT) staining, respectively. DAB staining was performed using a DAB substrate kit (Solarbio, Beijing, China), according to the manufacturer’s instructions. For NBT staining, wheat leaves were fully immersed in the 0.1 mg/mL NBT staining solution, vacuum-infiltrated for 30 min, and then incubated for 12 h. Subsequently, the leaves were decolorized in 95% (*v*/*v*) ethanol for 48 h. The stained leaves were photographed to assess H_2_O_2_ accumulation and superoxide (O^2−^) accumulation.

### 4.9. Determination of Antioxidant Enzyme Activity and Malondialdehyde (MDA) Content

Determination of the activities of superoxide dismutase (SOD), peroxidase (POD), and catalase (CAT) was carried out as described by Yue et al. [55]. Enzyme activities were expressed on a fresh weight basis (U g^−1^ FW) for relative comparisons among treatments. The Thiobarbituric Acid Reactive Substance (TBARS) content was determined using the thiobarbituric acid (TBA) method [51].

### 4.10. Haplotype Analysis and Salt Tolerance Evaluation of Haplotyped Materials

Population-scale variations for the *TaARF18* (*A/B/D*) coding sequence (CDS) regions were obtained from the 355 common wheat whole-genome sequencing (WGS) dataset in the Wheat Omics database (http://wheatomics.sdau.edu.cn/, accessed on 19 October 2025). Single nucleotide polymorphisms (SNPs) and (insertions–deletions) indels located within the *TaARF18* CDS regions were extracted and formatted for haplotype analysis. Haplotype classification, frequency summary, and visualization were performed by using TBtools (version 2.439).

Fourteen accessions were phenotyped under 150 mM NaCl salt stress to evaluate salt tolerance of haplotype-associated wheat lines. Shoot and root fresh weight, dry weight and lengths were recorded, and the activities of SOD, POD, and CAT were measured. Salt tolerance coefficients (STCs) were calculated for each trait, followed by normalization using the membership function method and integration with weight coefficients to obtain the D value (integrated salt tolerance index) for ranking overall salt tolerance [56].

### 4.11. Statistical Analysis

Sequence alignment and comparison of DNA and deduced amino acid sequences were conducted using GeneDoc (version 2.3.0.0). All statistical analyses and graphing were performed using GraphPad Prism (version 10.1.2). Data are presented as the mean ± SD. Statistical significance was determined using appropriate tests, as indicated in the figure legends, and significance levels were defined as * (*p* < 0.05), ** (*p* < 0.01), and *** (*p* < 0.001).

## 5. Conclusions

In this study, we cloned *TaARF18*, an auxin response factor, and investigated its role in response to salt stress. The coding sequences of *TaARF18-A*, *TaARF18-B*, and *TaARF18-D* were 2106, 2088, and 2088 bp in length, encoding 701, 695, and 695 amino acid residues, respectively. TaARF18 is a hydrophilic protein featuring typical Auxin-resp and B3 DNA-binding domains, and it exhibits relatively high evolutionary conservation among Poaceae species. The expression of *TaARF18* was upregulated in roots and leaves of wheat seedlings under salt stress. TaARF18 is localized in the nucleus. Silencing of *TaARF18* in wheat seedlings significantly enhanced their salt tolerance. Haplotype analysis according to the resequencing data of 355 wheat accessions revealed genetic variation in the *TaARF18* genomic region and identified 25, 31, and 16 haplotypes in *TaARF18-A*, *TaARF18-B*, and *TaARF18-D*, respectively. Fourteen wheat accessions with different haplotypes were evaluated under salt stress, and we found that *HapIII* of *TaARF18-A* exhibited the highest level of salt tolerance. Overall, our study provides a potential target for the genetic improvement of salt tolerance in wheat breeding.

## Data Availability

Data are contained within this article and the Supplementary Materials.

## Supplementary Materials

The following supporting information can be downloaded at https://www.mdpi.com/article/10.3390/plants15091375/s1: Figure S1. Hydrophilicity analysis and transmembrane domain prediction of TaARF18 proteins. (A–C) Hydrophilicity profiles of TaARF18-A, TaARF18-B, and TaARF18-D predicted using the Kyte–Doolittle method. (D–F) Prediction of transmembrane domains of TaARF18-A, TaARF18-B, and TaARF18-D proteins using TMHMM. Figure S2. Distribution of cis-acting elements in the 2000 bp upstream promoter regions of *TaARF18* homoeologs. Figure S3. Root scanning images of BSMV-mediated *TaARF18*-silenced and mock wheat seedlings after 12 days of salt treatment. Figure S4. Distribution of SNPs within the genomic regions of *TaARF18-A*, *TaARF18-B*, and *TaARF18-D*. Table S1. The *TaARF18* haplotypes of 14 wheat accessions. Table S2. Primers used in this study. Table S3. Genes identified in this study. Table S4. Haplotype of *TaARF18* homoeologs in 355 wheat accessions. Table S5. Membership function analysis (comprehensive salt-tolerance index). Table S6. Weight coefficients of physiological traits for the salt tolerance coefficient calculated using the membership function method.
