# Supplementary figures and images for: The Auxin Response Factor TaARF18-A Negatively Regulates Salt Tolerance in Common Wheat (Triticum aestivum L.)
Source: Plants (Basel). 2026 Apr 30;15(9):1375. doi: 10.3390/plants15091375 (PMC13164699; doi:10.3390/plants15091375)

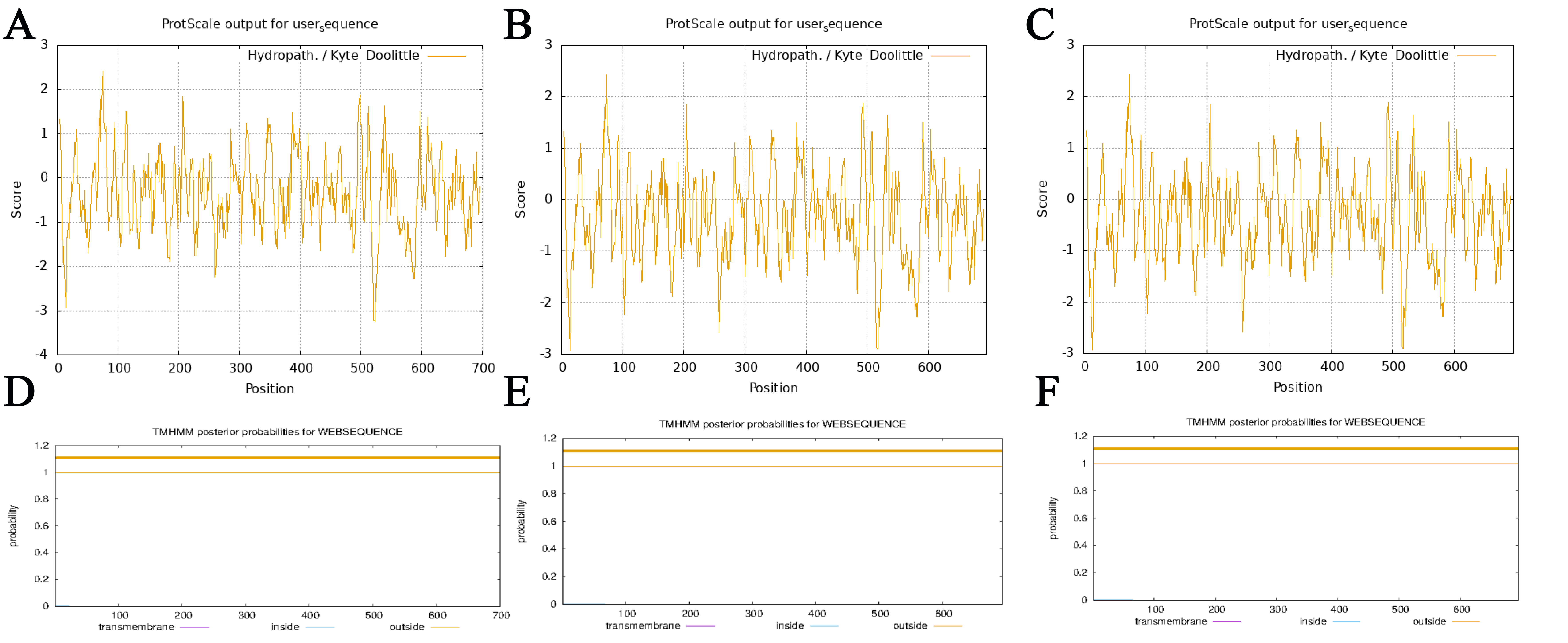

Supplement: Supplementary file 1 [file plants-15-01375-s001.zip › Figure S1.png]

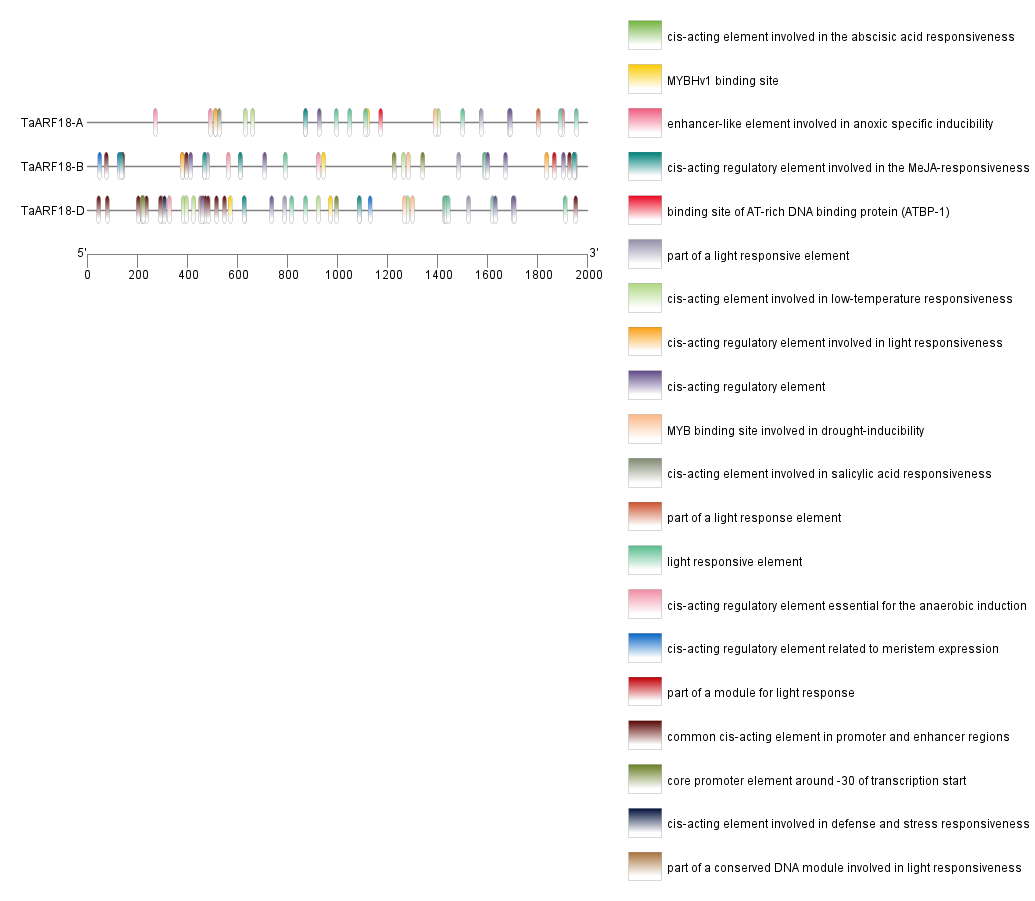

Supplement: Supplementary file 1 [file plants-15-01375-s001.zip › Figure S2.png]

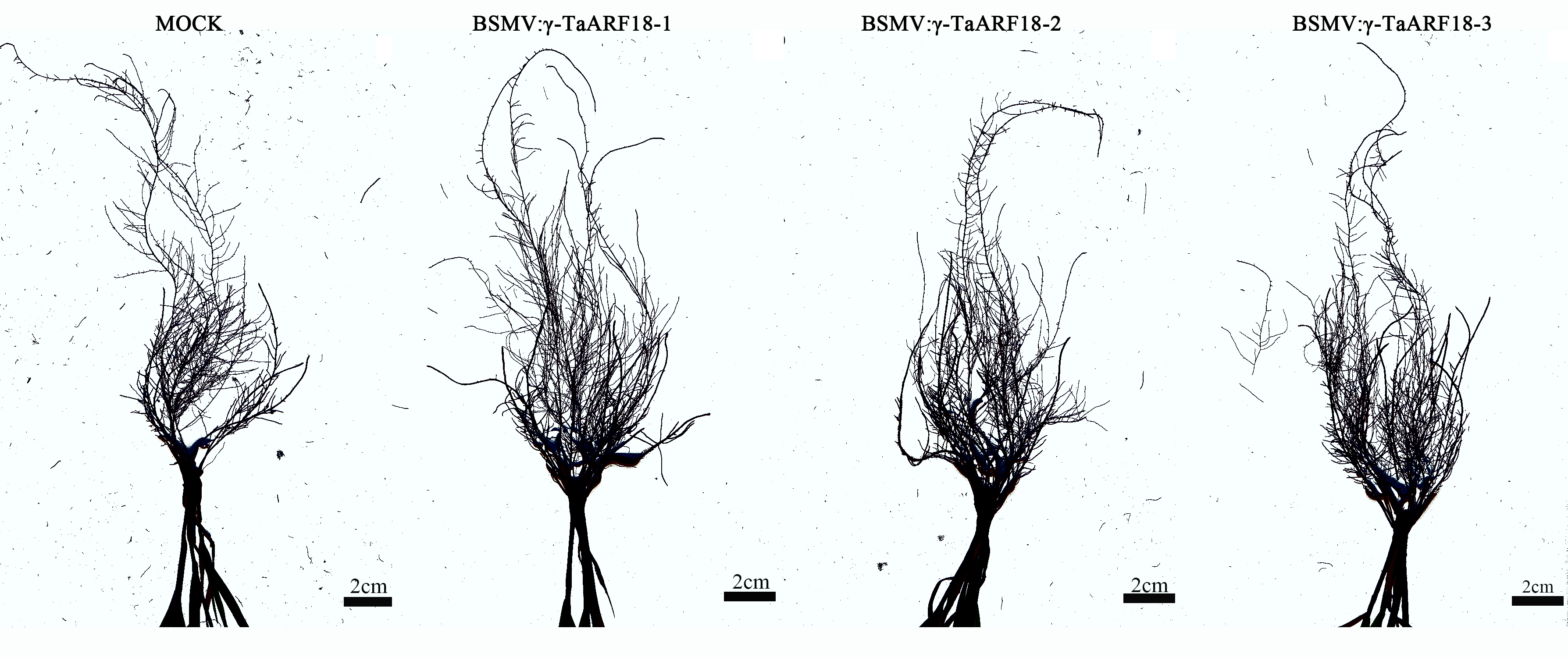

Supplement: Supplementary file 1 [file plants-15-01375-s001.zip › Figure S3.png]

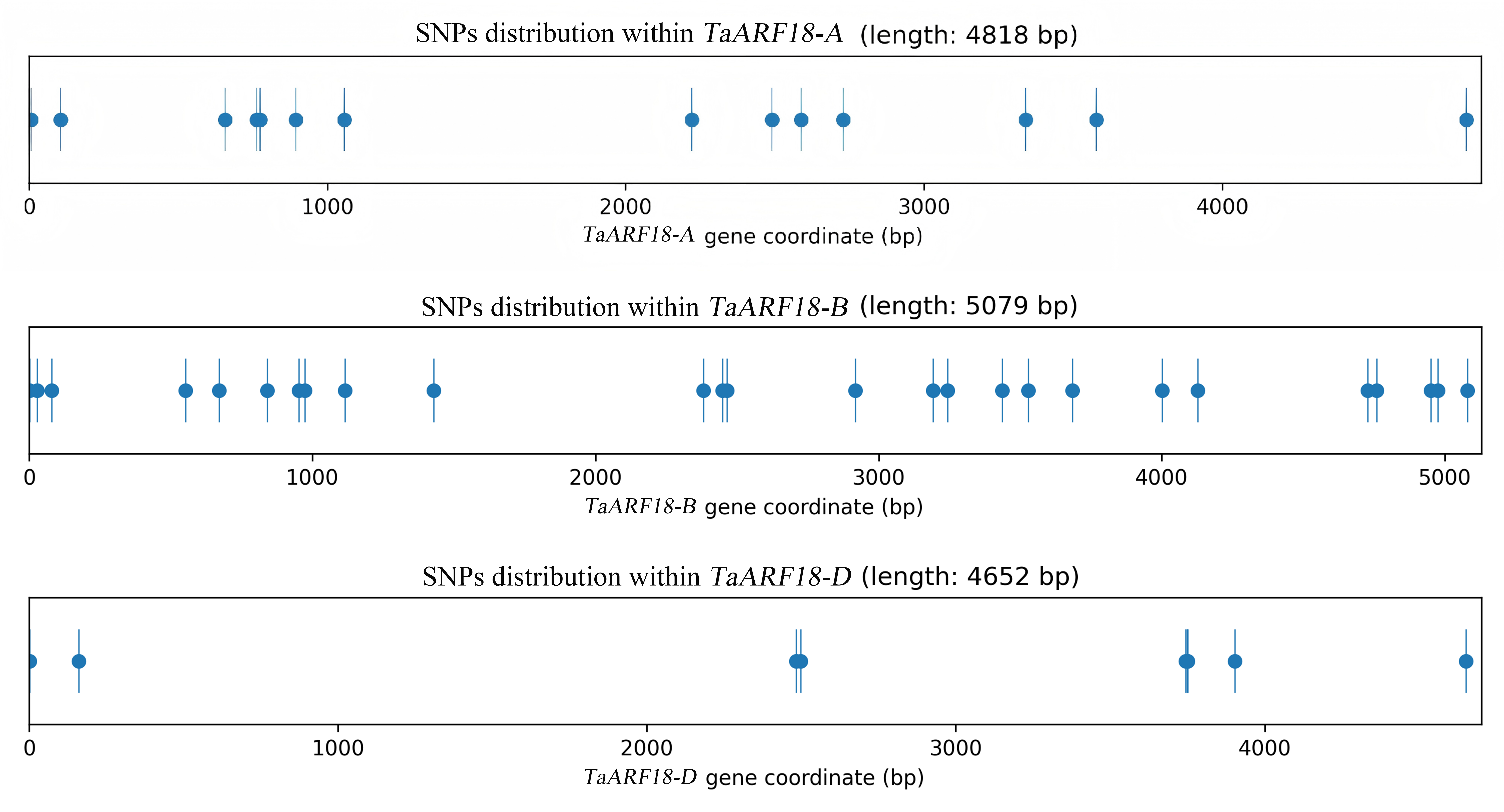

Supplement: Supplementary file 1 [file plants-15-01375-s001.zip › Figure S4.png]
